# ^68^Ga PSMA Uptake at Roux-en-Y Eso-jejunostomy Junction Mimicking the Recurrence of Gastric Carcinoma in PET/CT

**DOI:** 10.4274/mirt.galenos.2020.86729

**Published:** 2021-02-09

**Authors:** Esra Arslan, Tamer Aksoy, Merve Cin, Coşkun Çakır, Fadime Didem Can Trabulus, Tevfik Fikret Çermik

**Affiliations:** 1 University of Health and Sciences Turkey, İstanbul Training and Research Hospital, Clinic of Nuclear Medicine, İstanbul, Turkey; 2 University of Health and Sciences Turkey, İstanbul Training and Research Hospital, Department of Pathology, İstanbul, Turkey; 3 University of Health and Sciences Turkey, İstanbul Training and Research Hospital, Clinic of Surgery, Istanbul, Turkey

**Keywords:** 68Ga-PSMA, 18F-FDG, PET/CT, gastric carcinoma, prostate carcinoma

## Abstract

A 67-year-old male patient had undergone total gastrectomy and Roux-en-Y eso-jejunostomy 3 years ago for the treatment of tubular adenocarcinoma located at the corpus of the stomach. The patient was diagnosed with Gleason score 8 (4+4) metastatic prostate cancer during the follow-up period and received hormone therapy. Owing to his elevated prostate-specific antigen levels (77 ng/mL), his clinician referred him gallium-68 (^68^Ga) prostate-specific membrane antigen 11 (PSMA) positron emission tomography/computed tomography (PET/CT) for restaging. PET/CT showed multiple ^68^Ga PSMA receptor-positive skeletal lesions and linear PSMA activity at the eso-jejunostomy junction. He was then referred to undergo ^18^fluorine-fluorodeoxyglucose (^18^F-FDG) PET/CT to screen for gastric carcinoma recurrence. PET/CT images demonstrated no ^18^F-FDG avid lesion. However, endoscopy and biopsy performed with samples from the eso-jejunostomy junction revealed superficial benign squamous epithelial fragments.

## Abstract (tr)

Altmış yedi yaşında erkek hastaya mide korpusunda tespit edilen tubular adenokarsinom nedeniyle 3 yıl önce total gastrektomi ve Roux-en-Y oeso-jejunostomi yapılmıştır. Takip süresince hasta Gleason skoru 8 (4+4) metastatik prostat kanseri tanısı da alarak hormonoterapi uygulandı. Prostat spesifik antijen düzeylerinde (77 ng/mL) yükselme saptanması nedeniyle yeniden evreleme amaçlı galyum-68 prostat spesifik membran antijeni 11 (PSMA) pozitron emisyon tomografi/bilgisayarlı tomografi (PET/BT) görüntüleme için hasta kliniğimize refere edildi. ^68^Ga PSMA PET/BT görüntülemesinde oeso-jejunostomi kavşağında lineer PSMA aktivitesi ve multipl PSMA reseptör pozitif iskelet lezyonları saptandı. Gastrik karsinomun nüksü şüphesi nedeni ile hasta ^18^F-FDG PET/BT incelemesi açısından yeniden kliniğimize sevk edildi. ^18^F-FDG PET/BT görüntülerinde FDG pozitif malign prosesi düşündürebilecek lezyon görülmedi. Oeso-jejunostomi anastomoz hattından yapılan endoskopi ve biyopsi incelemesinde yüzeyel benign skuamöz epitelyal fragmanlar saptandı.

## Figures and Tables

**Figure 1:**
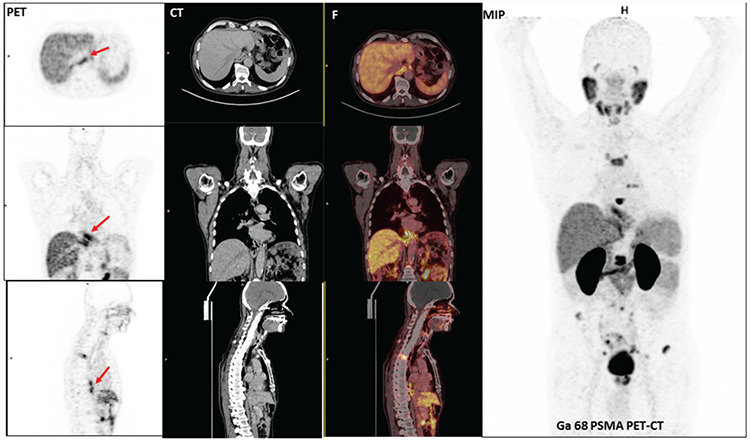
Gallium-68 (^68^Ga) prostate-specific membrane antigen 11 (PSMA) positron emission tomography/computed tomography (PET/CT) shows increased uptake at multiple metastatic skeletal lesions in the vertebral column and pelvic bones as well as linear PSMA accumulation at the esojejunostomy line (red arrows). PSMA is a type 2 transmembrane protein that acts as a glutamate carboxypeptidase enzyme (1,2). Owing to its high expression in prostate cancer cells, PSMA is often used conveniently as a target for diagnostic and therapeutic purposes in nuclear medicine. Normal ^68^Ga PSMA uptake might be seen in the following structures, with descending avidity: Kidneys (8 times higher than hepatic uptake), submandibular glands, parotid glands (3 times higher than hepatic uptake), descending duodenum, lacrimal glands, spleen, descending colon, Waldeyer ring in the neck, vocal cords, liver, and rectum (3). In case of benign lesions, most ^68^Ga PSMA uptake is of low intensity or non-focal, with some notable exceptions (e.g., cutaneous, vertebral, and hepatic hemangiomas) exhibiting prominent uptake (4). Prostate cancer commonly spreads to the bones and lymph nodes. Although the spread of prostate cancer to the gastrointestinal tract is very rare, the possibility of metastasizing to the stomach should be kept in mind when a patient presents with gastrointestinal symptoms or hemorrhage (5). A few reports have demonstrated prostate carcinoma metastases in the stomach (5,6,7,8). A study by Shetty et al. (9) reported mild PSMA uptake in the gastric cardia in a case of high-grade invasive gastric adenocarcinoma. In another study, they found PSMA to be expressed by endothelial cells in keloids, granulation tissue from heart valves and pleura, and different phases of the cycling endometrium. It was reported that PSMA was not expressed by endothelium associated with Barrett’s mucosa, even in the presence of associated dysplasia (10). It should be kept in mind that patients should be evaluated individually as PSMA uptake might be seen in both benign and malignant lesions.

**Figure 2:**
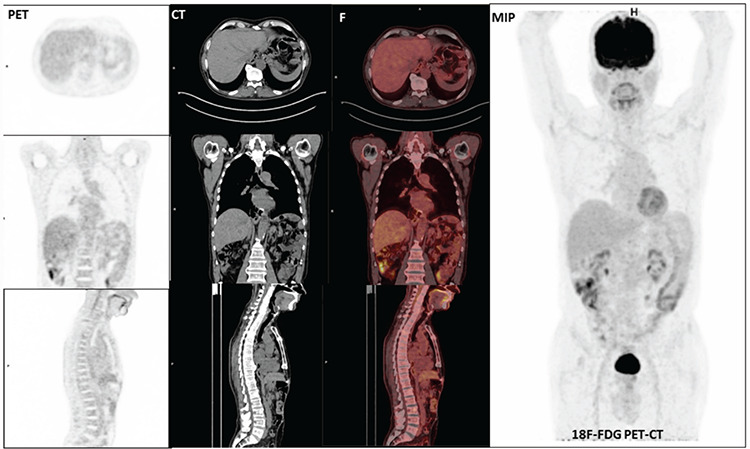
No abnormal ^18^fluorine-fluorodeoxyglucose (^18^F-FDG) uptake at the eso-jejunostomy line was detected in ^18^F-FDG PET/CT computed tomography images.

**Figure 3:**
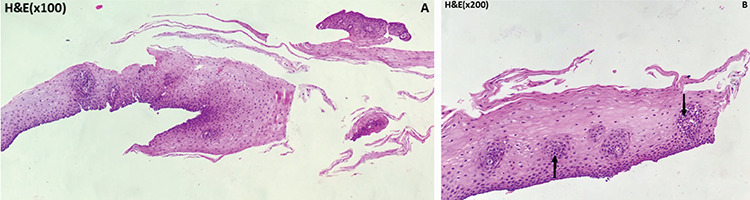
Owing to the suspicious PSMA 11 uptake at the eso-jejunostomy line, endoscopy and biopsy were performed with samples from this line. Benign squamous epithelial fragments stained with hematoxylin and eosin (H and E) with 100 times magnification (A). Black arrow shows benign squamous epithelial fragments stained with H and E with 200 times magnification (B).
